# Development of Artificial-Neural-Network-Based Permanent Deformation Prediction Model of Unbound Granular Materials Subjected to Moving Wheel Loading

**DOI:** 10.3390/ma15207303

**Published:** 2022-10-19

**Authors:** Wenjun Hua, Qunding Yu, Yuanjie Xiao, Wenqi Li, Meng Wang, Yuliang Chen, Zhiyong Li

**Affiliations:** 1 School of Civil Engineering, Central South University, Changsha 410075, China; 2 Urban Rail and Underground Engineering Design and Research Institute, China Railway Siyuan Survey and Design Group Co., Ltd., Wuhan 430063, China; 3 MOE Key Laboratory of Engineering Structures of Heavy Haul Railway, Central South University, Changsha 410075, China; 4 Hunan Communications Research Institute Co., Ltd., Changsha 410015, China

**Keywords:** unbound granular materials, cyclic triaxial test, permanent deformation, prediction model, artificial neural network (ANN)

## Abstract

The majority of existing regression models for unbound granular materials (UGMs) consider only the effects of the number of loading cycles and stress levels on the permanent deformation characteristics and are thus unable to account for the complexity of plastic deformation accumulation behavior influenced by other factors, such as dry density, moisture content and gradation. In this study, research efforts were made to develop artificial-neural-network (ANN)-based prediction models for the permanent deformation of UGMs. A series of laboratory repeated load triaxial tests were conducted on UGM specimens with varying gradations to simulate realistic stress paths exerted by moving wheel loads and study permanent deformation characteristics. On the basis of the laboratory testing database, the ANN prediction models were established. Parametric sensitivity analyses were then performed to evaluate and rank the relative importance of each factor on permanent deformation behavior. The results indicated that the developed ANN prediction model is more accurate and reliable as compared to previously published regression models. The two major factors influencing the magnitude of accumulated plastic deformation of UGMs are the shear stress ratio (SSR) and the number of loading cycles, of which the calculated influence coefficients are 38% and 41%, respectively, while the degree of influence of gradation is twice that of the confining pressure.

## 1. Introduction

As the core layer of the pavement/track foundation, the base/subbase course comprising unbound granular materials (UGMs), such as unbound aggregates [1], construction and demolition waste (CDW) [2] and granite scraps [3], is directly subjected to the repeated applications of moving wheel loads, thus significantly affecting the deformation behavior of such UGMs as well as the field performance of pavement systems incorporating such unbound layers. Even if the subgrade has sufficient bearing capacity, permanent deformation or rutting would occur if the moving load is large enough or the loading period is long enough, thus reducing the service life of the pavement system. It was found that permanent deformation of unbound granular layers could account for up to 50% of the pavement surface rut in severe cases, e.g., Ref [4]. Excessive permanent deformation could result in corresponding structural or functional damage to overlying pavement structures and thus reduce the long-term stability, operation safety and ride comfort. Therefore, it is of great significance to study the influencing factors, predictive models, as well as control indices of permanent deformation of unbound granular materials.

There are several test methods to evaluate the cumulative plastic deformation of various types of soils by replicating the realistic in situ stress conditions, among which the cyclic triaxial test is widely used for unbound granular pavement materials. Previous studies were conducted to investigate the effects of influencing factors, such as the material type [5], aggregate gradation [6], moisture content [7,8], dry density [7,9], stress level [7,9], and loading frequency [8,10] and waveform [11] on the deformation characteristics of UGMs through laboratory repeated load triaxial tests. Among those factors, the gradation was found to exhibit the greatest influence on the bearing capacity and stability of such UGMs [12]. For instance, Nguyen [13] studied, from laboratory large-scale triaxial tests, the factors influencing the permanent deformation resistance of graded aggregates and reported that the aggregates with plastic fines exhibited the greatest accumulated plastic strain. Ghabchi [14] conducted laboratory repeated load triaxial tests on graded aggregates at low confining pressure levels and found that the accumulated plastic strain of mono-sized aggregates was the greatest under the same stress condition. Cai [15] carried out several laboratory repeated load triaxial tests on unbound graded aggregate materials and found that the gradation had a great influence on permanent deformation under different loading conditions. However, the above-surveyed studies only investigated the strength and deformation characteristics of crushed aggregate fill materials with typical gradations, thus lacking a broader coverage and representation of UGMs. With the development of gradation models based on fractal theory and particle packing theory, the existing research efforts have focused on quantifying the gradation into continuous values to better establish a quantitative relationship between gradation and mechanical properties. These gradation models can quantify the variation of soil properties over a specified range of gradations and could be applied to design aggregates with desirable mechanical properties.

Empirical regression models, one of the most popular methods for estimating the permanent deformation of subgrade soil, can accurately describe the mechanical properties of materials based on a large number of laboratory test results while requiring fewer parameters and being simple to apply in practical engineering. These models are used to describe the relationship between cumulative plastic strain and factors such as the number of loading cycles [16], stress level [17,18], loading frequency [19], soil strength [20,21] and water content [22,23] by regression analysis, which has the advantage of simple formulations and fast calculating speeds. However, developing regression models requires specifying the relationships between the above parameters in advance and evaluating many linear and nonlinear equations. Although many regression models have been purposed [10], there are few nonlinear models with more than two independent variables that can accurately describe the complex permanent deformation characteristics of UGMs under the impact of factors such as gradation.

Recently, artificial neural network (ANN) models have been successfully applied to quantitatively predict the parametric properties of different pavement materials, e.g., Refs [24,25,26]. The neural network is a kind of machine-learning algorithm and is capable of modeling the complex relationships between multiple variables by simulating biological neurons [27]. A conventional artificial neural network is composed of the input layer, the hidden layers and the output layer. Each layer contains a certain number of neurons, which are linked to neurons in the preceding or subsequent layer via an activation function. Based on the error back-propagation mechanism, the neural network constantly adjusts the weights and biases from one layer to the next, eventually making the error between the predicted and expected values less than a predetermined threshold. Artificial-neural-network (ANN)-based models are increasingly applied to reveal the underlying complex relations (whose explicit functions are generally not available) due to their robustness and simplicity. Mollahasani et al. [28] developed an ANN model to estimate the cohesion of soils by using a series of unconsolidated–undrained triaxial test data, which exhibited better predictive performance than regression models. Nazzal and Tatari [29] also reported that the ANN-based model of subgrade soil has higher prediction accuracy than regression models. Kim et al. [30] developed an ANN model for estimating the resilient modulus of subgrade soils from their physical properties and applied stress state. Similarly, Saha et al. [31] developed an ANN model for coarse-grained soils to predict the resilient modulus from their physical properties with the coefficient of determination (R^2^) greater than 0.9. Ghorbani et al. [32] evaluated the influences of the number of load applications, the content of recycled asphalt, temperature and deviatoric stress on the accumulated plastic deformation of recycled Portland cement concrete and recycled asphalt mixtures by using an ANN model, respectively. Ullah et al. [33] also used an ANN model to evaluate the influence of binder content on the permanent deformation of recycled asphalt mixtures. Although the above-mentioned studies in the literature covered certain factors influencing the permanent strain of bounded materials by developing ANN-based models, the application of ANN-based models to predict the permanent deformation of unbound graded aggregate materials with varying permeable gradations under different stress levels has rarely been studied, to the authors’ best knowledge.

In this study, five distinct permeable gradations were initially designed using the gravel-to-sand ratio (*G*/*S*) index based on the particle packing theory, and the effect of gradation on the permanent deformation of unbound graded aggregate materials was investigated by conducting cyclic triaxial tests at various shear stress ratio (SSR) levels. A nonlinear regression model was then developed using the cumulative plastic strain data obtained from the tests. The number of loading cycles, deviatoric stress and shear stress ratio were used as independent variables to predict the permanent deformation of unbound graded aggregate materials. Subsequently, the *G*/*S* index was included as an input variable to build a well-structured artificial neural network, and sensitivity analysis was performed to determine how much each input variable affected the output permanent deformation. Finally, the prediction accuracy and robustness of these two methods were thoroughly discussed. It is possible to apply the validated ANN-based prediction model to assist the mechanistic study and design of pavement.

## 2. Materials and Methods

### 2.1. Materials Tested

The test material was gravel from a quarry in the suburbs of Changsha city, whose parent rock is limestone. During the tests, the coarse particles with particle size greater than 2.36 mm were first washed and dried according to the relevant specifications for highway subgrade [34] and sieved by using square-hole sieves with opening sizes of 26.5 mm, 16 mm, 9.5 mm, 4.75 mm and 2.36 mm. Meanwhile, the fine particles with particle sizes smaller than 2.36 mm were sieved using sieve opening sizes of 0.6 mm and 0.075 mm. Subsequently, the gradations tested in this study were properly designed and controlled by the gravel-to-sand ratio (*G*/*S*) parameter proposed previously by the authors [35,36,37,38]. In fact, the *G*/*S* parameter can be calculated from Equations (1) and (2).

(1) $$G/S=\frac{1-P_{4.75}}{P_{4.75}-P_{0.075}}=\frac{D_{\max}^{n}-4.75^{n}}{4.75^{n}-0.075^{n}}$$

(2) $$P_{i}={\left(\frac{d_{i}}{D_{\max}}\right)}^{n}$$

where $P_{i}$ is the percentage passing by weight; $d_{i}$ is the sieve diameter (mm); *D*_max_ is the largest particle diameter (mm); *n* is the parameter determining the shape of gradation curves.

**Figure 1:**
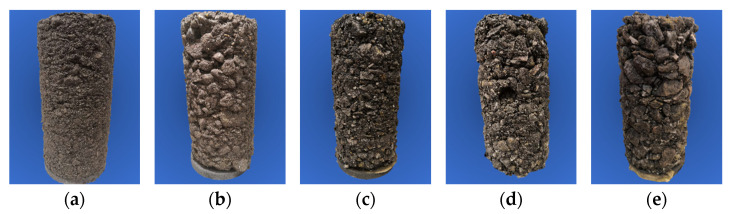
Pictures of unbound aggregate specimens with various gradations: (**a**) *G*/*S* = 1.0; (**b**) *G*/*S* = 1.6; (**c**) *G*/*S* = 1.8; (**d**) *G*/*S* = 2.0; (**e**) *G*/*S* = 2.5.

**Figure 2:**
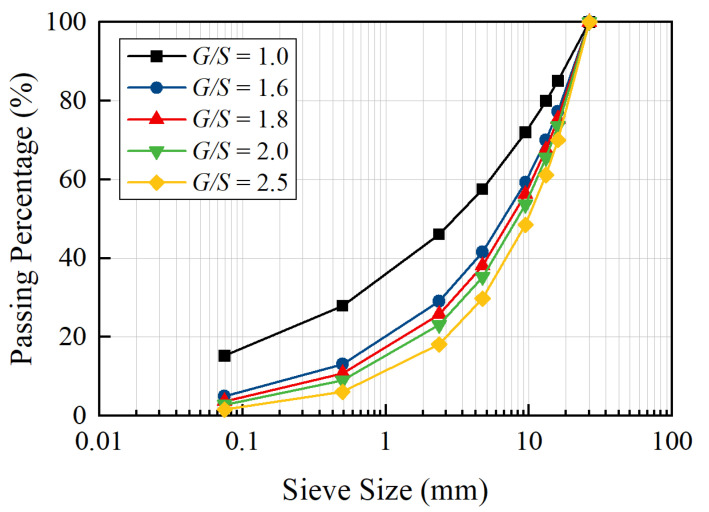
Gradation curves of unbound aggregate base materials tested.

In this study, a total of five groups of specimens with different gradations were designed, as shown in Figure 1. The gradation curves are shown in Figure 2, and the relevant parameters are shown in Table 1.

### 2.2. Testing Apparatus

**Figure 3:**
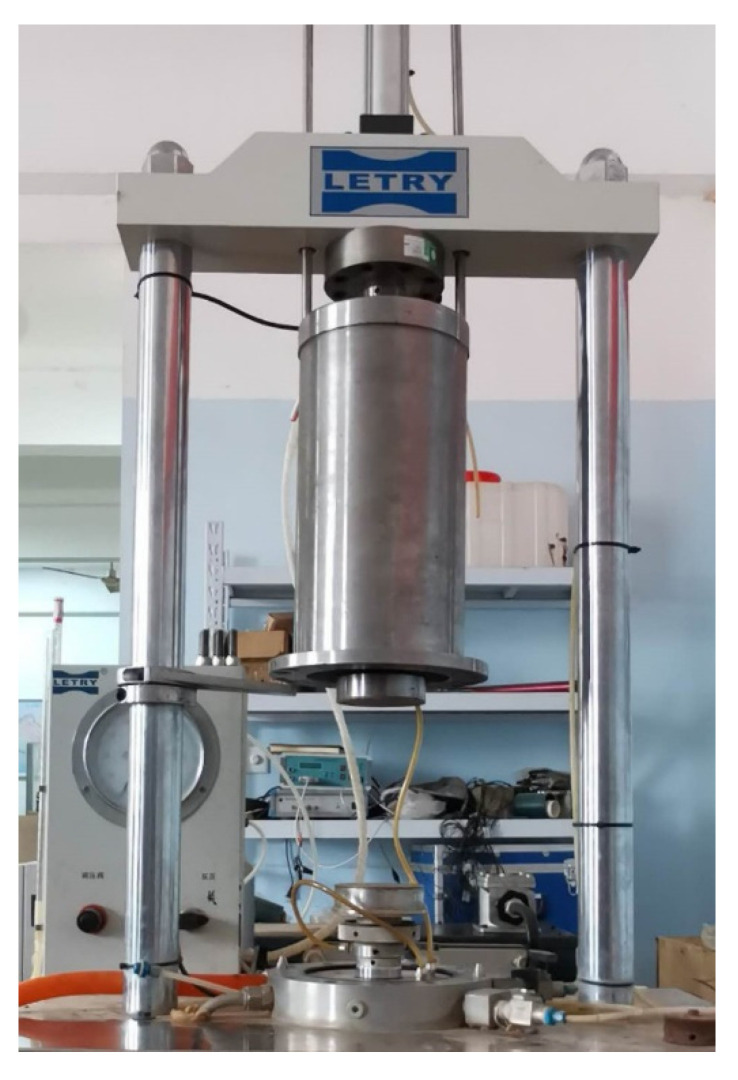
The close-up photo of the triaxial apparatus used for laboratory monotonic and cyclic triaxial tests.

The LETRY-STD triaxial apparatus, as shown in Figure 3, mainly consists of the axial loading system, confining pressure control system, pressure chamber lifting mechanism and motor control and data acquisition system. Among them, the axial loading system is composed of transducers for axial force and displacement, and the base of the pressure chamber has a water inlet, a water outlet and a pressure measurement interface. The pressure chamber lifting mechanism comprises a lifting frame, a direction control valve and connecting pipelines.

### 2.3. Cyclic Triaxial Tests

**Figure 4:**
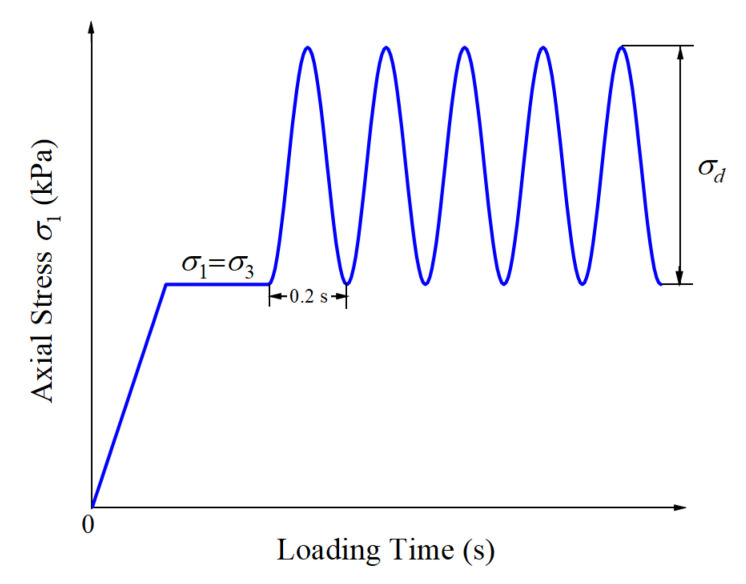
The time–history curve of the axial stress.

The laboratory repeated load triaxial tests were conducted under consolidated drained conditions to characterize the plastic axial strain accumulation of unbound graded aggregate materials with different gradations subjected to various stress states. The specific testing matrix is shown in Table 2. Note that the shear strength parameters, i.e., the internal friction angle and the apparent cohesion, were determined from laboratory monotonic triaxial compression tests under consolidated drained conditions according to the Chinese standard (JTG 3430-2020) [39]. The cylindrical specimens were fabricated in a split mold with 100 mm inner diameter and 200 mm height and then subjected to laboratory repeated load triaxial tests by following the AASHTO-T307 testing protocol. To be specific, the entire loading process consisted of the initial pre-loading conditioning stage and the subsequent applications of repeated loading [40]. The triaxial specimens were conditioned for 1000 load applications with the combination of 103.4 kPa confining pressure and 103.4 kPa deviatoric stress in order to eliminate contact deformation between the loading cap (or base) and the specimens, followed by up to 50,000 repeated load applications. The goal was to simulate the long-term deformation behavior of the tested materials. The haversine loading waveform applied during the tests is shown in Figure 4, whose loading frequency was set to 5 Hz. It should be noted that there was actually no rest period between two adjacent load applications, as limited by the triaxial apparatus adopted in this study (i.e., only continuous waveform can be applied), which, however, needs to be properly taken into account in future studies.

The 60 triaxial specimens were prepared according to the same degree of compaction (i.e., the ratio of achieved dry density to the maximum dry density under the same compaction energy level) of 0.9 and the respective optimum moisture contents. Both the maximum dry density and the optimum moisture content values of unbound aggregates with different gradations were determined from the laboratory compaction tests according to the Chinese standard (JTG 3430-2020) [39]. Note that the adopted compaction procedure is similar to that of the modified Proctor compaction (AASHTO T180). During the compaction tests, the unbound aggregates with designated gradations and moisture contents were placed into a mold with 152 mm inner diameter and 120 mm height in three sublayers. Each of the three sublayers was then compacted by 98 blows of a 4.5 kg hammer falling freely from a height of 450 mm, thus applying a compaction energy per unit volume of 2691 kJ/cm^3^. The measured compaction curves of gravitational moisture content versus achieved dry density were skipped for brevity, while the obtained results of maximum dry density and optimum moisture content are listed in Table 2.

**Figure 5:**
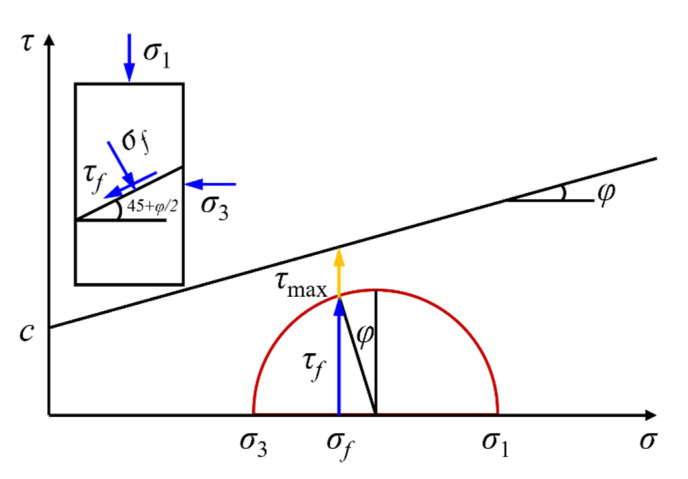
Illustration of the definition of shear stress ratio (SSR).

Depending on the driving speed, traffic density, soil parameters, depth of the subgrade and pavement structure, the amplitude and frequency of dynamic stresses generated within the subgrade of urban roads or highways under traffic loads are different. The confining pressure experienced in unbound pavement base/subbase courses typically ranges from 12.64 to 99.13 kPa [15,41,42]. Therefore, three representative levels of confining pressure were selected for use in this study, i.e., 50 kPa, 100 kPa and 150 kPa. Note that the relatively higher confining pressure level selected in this study was partly due to the equipment limitation and was meant to mimic the engineering applications where either premium base/subbase courses are constructed to sustain heavy traffic loading or unsurfaced low-volume roads with unbound granular base/subbase courses are encountered. The shear stress ratio (SSR) was employed to control the deviatoric stress value $\sigma_{d}$, and four typical stress levels (the SSR values were 0.3, 0.5, 0.7 and 0.9, respectively) were designed. As shown in Figure 5, the SSR value is defined by the Mohr–Coulomb criterion as the ratio of shear stress to shear strength, which can be calculated according to Equations (3)–(6).

(3) $$\mathrm{SSR}=\frac{\tau_{f}}{\tau_{max}}$$

(4) $$\tau_{f}=\frac{\sigma_{d}cos\varphi }{2}$$

(5) $$\tau_{max}=\sigma_{f}tan\varphi +c$$

(6) $$\sigma_{f}=\sigma_{3}+\frac{\sigma_{d}\left(1-sin\varphi \right)}{2}$$

where $\tau_{f}$ is the shear stress on the shear plane when shear failure occurs; $\tau_{max}$ is the shear strength calculated according to the Mohr–Coulomb criterion; $\sigma_{d}$ denotes the deviatoric stress applied; φ is the internal friction angle;$\;\sigma_{f}$ is the normal stress on the shear plane when shear failure occurs; $\sigma_{3}$ is the confining pressure; c is the apparent cohesion.

## 3. Testing Results and Analysis

### 3.1. Effect of Shear Stress Ratio on the Cumulative Plastic Strain

**Figure 6:**
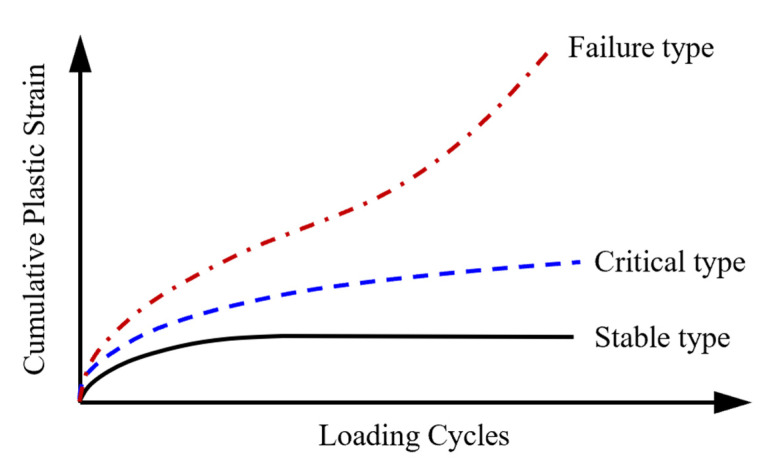
Schematic diagram of different permanent deformation behaviors of unbound aggregate base materials.

According to their varying tendencies, the axial cumulative plastic strain–loading cycle curves of unbound granular materials can be classified into three types (i.e., stable, critical and failure type) [43], as shown in Figure 6. The curve of the stable type shows that the growth rate of plastic strain gradually decreases with the increase in the number of loading cycles and eventually rarely changes, reflecting the situation where the specimen is gradually compacted under the action of dynamic loading. The curve of the failure type demonstrates that, as the number of loading cycles increases, so does the growth rate of plastic strain. Before the sample is compacted during the triaxial test, shear failure occurs under a small number of loading cycles. The curve of the critical type lies between the curves of the stable type and failure type, suggesting that, as the number of cycles increases, the accumulating speed of plastic strain gradually declines and finally stabilizes. This situation reflects the fact that the particle rearrangement in the initial stage enhances the stability of the particle skeleton, whereas the compression of air voids and shear deformation cause permanent deformation.

**Figure 7:**
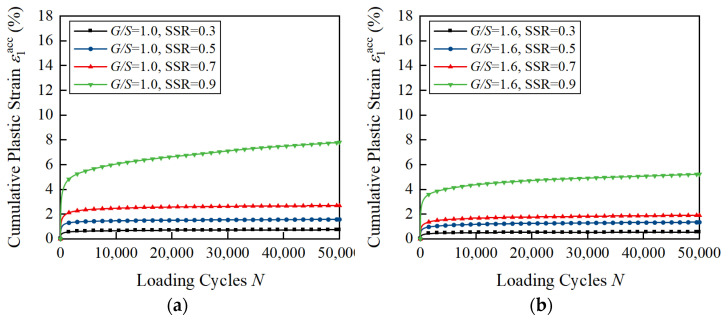

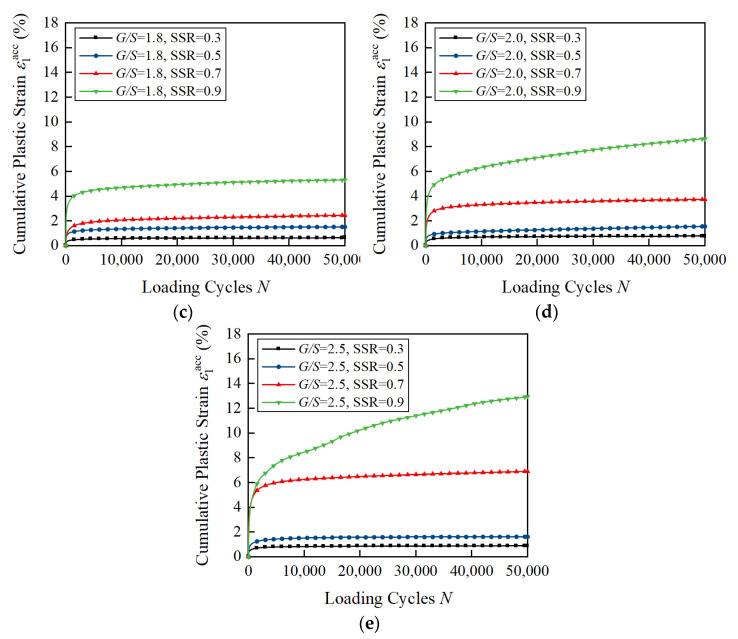
Laboratory-measured permanent deformation of unbound aggregate base materials with different *G*/*S* values under confining pressure of 150 kPa and different SSR levels: (**a**) *G*/*S* = 1.0; (**b**) *G*/*S* = 1.6; (**c**) *G*/*S* = 1.8; (**d**) *G*/*S* = 2.0; (**e**) *G*/*S* = 2.5.

Figure 7 shows the axial cumulative plastic strain $\varepsilon_{1}^{\mathrm{acc}}$ of unbound aggregate base materials with different *G*/*S* values under a confining pressure of 150 kPa and varying SSR levels as a function of the number of loading cycles. It can be seen that, as the shear stress ratio increases, the development trend of the cumulative plastic strain of unbound aggregate base materials with different *G*/*S* values shifts from the stable type to the critical type. When SSR < 0.7, the cumulative plastic strain curves become gentle, while the number of loading cycles increases. All the cumulative plastic strain curves can be classified as stable type, which shows that the cumulative plastic strain grows rapidly when *N* < 10,000, and the growth rate of the cumulative plastic strain gradually slows down after *N* > 10,000 and finally becomes stable. When SSR > 0.7, the growth rate of the cumulative plastic strain is relatively fast when *N* < 10,000 and then gradually slows down, but it does not tend to be stable, and the cumulative plastic strain curves can be classified as critical type. It is noteworthy that the growth rate of the cumulative plastic strain of unbound aggregate base materials with *G*/*S* = 2.0 and *G*/*S* = 2.5 at this SSR level still shows an increasing trend at the later stage of loading, indicating that the curves may develop into failure type. Due to the small initial compactness of the specimens (the degree of compaction of all the specimens is 0.9), the trend can only appear after a larger number of loading cycles. This indicates that the stress level not only influences the magnitude and growth rate of permanent deformation but also the accumulation pattern of permanent deformation (i.e., plastic shakedown, plastic creep or incremental collapse).

### 3.2. Effect of Confining Pressure on the Cumulative Plastic Strain

**Figure 8:**
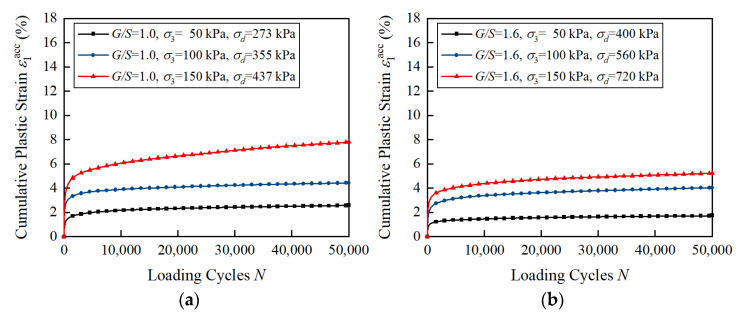

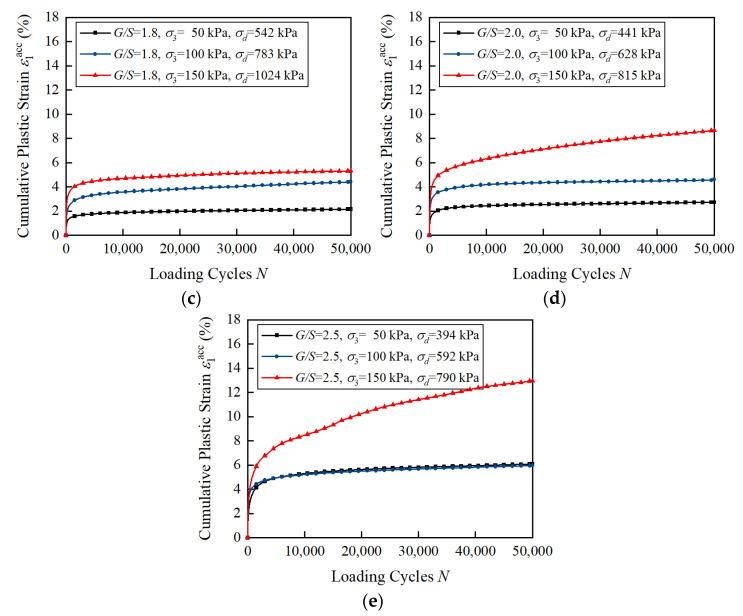
Laboratory-measured permanent deformation of unbound aggregate base materials with different *G*/*S* values under SSR = 0.9 and different confining pressure levels: (**a**) *G*/*S* = 1.0; (**b**) *G*/*S* = 1.6; (**c**) *G*/*S* = 1.8; (**d**) *G*/*S* = 2.0; (**e**) *G*/*S* = 2.5.

Figure 8 shows the curves of axial cumulative plastic strain $\varepsilon_{1}^{\mathrm{acc}}$ versus the number of loading cycles of unbound aggregate base materials with different *G*/*S* values under SSR = 0.9 and different confining pressure levels. For the UGMs with the same *G*/*S* value, it is evident that the cumulative plastic strain increases with the increase in confining pressure. This is related to the fact that the dynamic deviatoric stress increases as the confining pressure increases at the same SSR level. Therefore, it is necessary to consider both the shear stress ratio and confining pressure to analyze the development law of the cumulative plastic strain of unbound aggregate base materials.

**Figure 9:**
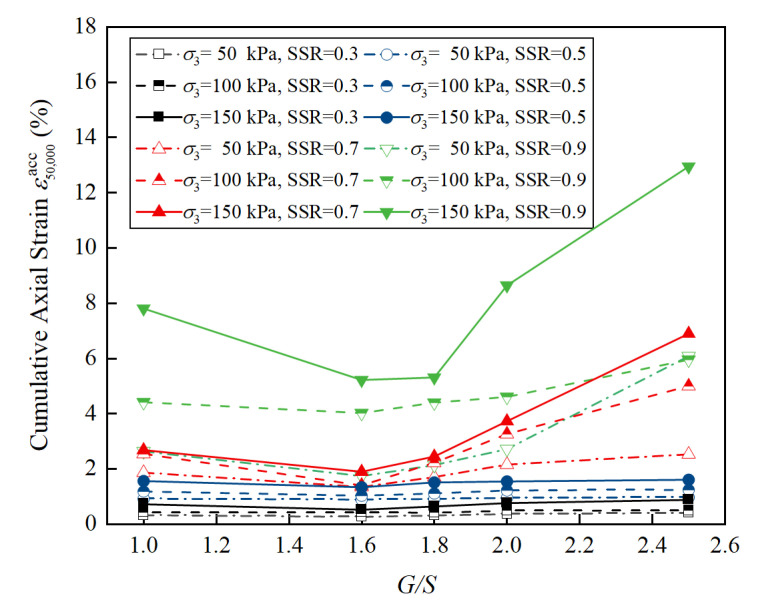
Laboratory-measured cumulative plastic strain at *N* = 50,000 versus *G*/*S* values of specimens.

In order to further investigate the impact of confining pressure on the permanent deformation of unbound aggregate base materials under different shear stress levels, the relation curves between the axial cumulative plastic strain at the number of loading cycles *N* = 50,000 and *G*/*S* values were obtained for 60 sets of specimens, as plotted in Figure 9. It can be concluded that the confining pressure has a more pronounced influence on the accumulated plastic strain of unbound aggregate base materials with identical *G*/*S* values at higher shear stress ratio (SSR) levels. For example, when the confining pressure was 50 kPa, 100 kPa, 150 kPa and SSR = 0.3, the cumulative plastic strains of specimens with *G*/*S* = 2.0 were 0.38%, 0.50% and 0.78%, respectively. When SSR ≥ 0.7, the confining pressure has a significant effect on the cumulative plastic strain of unbound aggregate base materials with the same *G*/*S* value. For example, when the confining pressure was 50 kPa, 100 kPa, 150 kPa and SSR = 0.9, the cumulative plastic strains of the specimens with *G*/*S* = 2.0 were 2.73%, 4.62% and 8.66%, respectively.

### 3.3. Effect of Gradation on the Cumulative Plastic Strain

**Figure 10:**
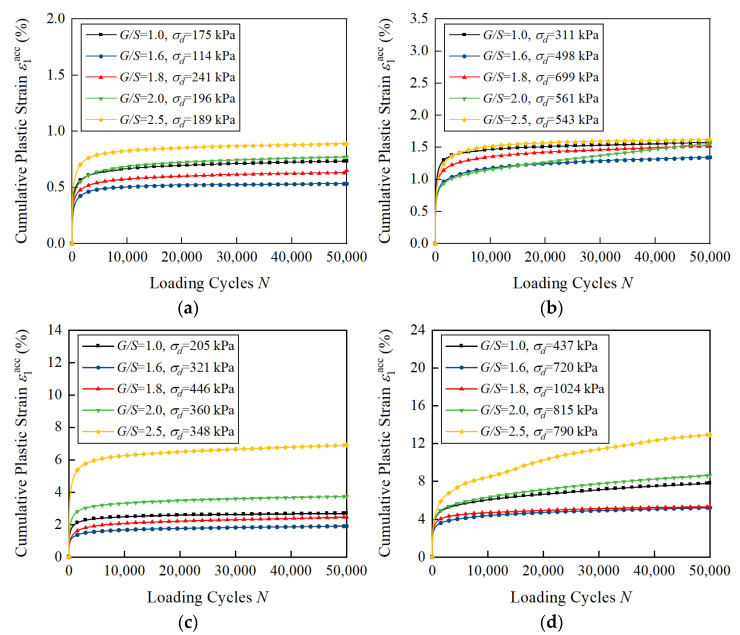
Laboratory-measured permanent deformation of unbound aggregate base materials with different *G*/*S* values under confining pressure of 150 kPa: (**a**) SSR = 0.3; (**b**) SSR = 0.5; (**c**) SSR = 0.7; (**d**) SSR = 0.9.

Figure 10 shows the curves of the axial cumulative plastic strain $\varepsilon_{1}^{\mathrm{acc}}$ versus the number of loading cycles of unbound aggregate base materials with different *G*/*S* values under a confining pressure of 150 kPa and different SSR levels. It can be seen that, under the same confining pressure but different SSR levels, the accumulated plastic axial strain values of unbound aggregate materials with various *G*/*S* values are observed to ascend in the following order: *G*/*S* = 1.6 < *G*/*S* = 1.8 < *G*/*S* = 1.0 < *G*/*S* = 2.0 < *G*/*S* = 2.5. This result is actually consistent with that reported by Cai [15], i.e., the accumulated plastic axial strain values of UGMs with an uneven skeleton-void structure (*C*_u_ = 22.8) are the smallest for varying cyclic stress magnitudes, followed by UGMs with a suspended skeleton structure (*C*_u_ = 19.6) and skeleton structure (*C*_u_ = 6.1). The internal packing structures of unbound aggregate specimens play an important role in determining their resistance to plastic deformation accumulation. It should be noted that the specimens with high shear strength (*G*/*S* = 1.8) do not have the best performance in resisting permanent deformation, while the specimens with poor gradation (*G*/*S* = 1.0) and low shear strength still have good resistance to plastic deformation. The accumulated plastic axial strain curves corresponding to specimens with various *G*/*S* values show little differences when SSR ≤ 0.5, while such curves show greater differences when SSR ≥ 0.7. This trend was also reported by Cai [15], i.e., that the maximum difference of $\varepsilon_{1}^{\mathrm{acc}}$ among different UGMs was about 0.029%, 0.075% and 0.208% under a 20 kPa confining pressure and different deviator stress levels of 20 kPa, 60 kPa and 100 kPa, respectively. This indicates that the influence of gradation on the accumulated plastic axial strain of unbound aggregate base materials becomes more significant with increasing shear stress ratio. This is actually consistent with the conclusion drawn by Qamhia [6] that the effect of gradation on permanent strain accumulation was significantly more pronounced at high shear stress ratios as compared to that at low shear stress ratios.

## 4. Development of the Permanent Deformation Prediction Model

### 4.1. Development of Empirical Regression Model

Most of the existing regression models of permanent deformation only use the number of loading cycles as a single parameter, as shown in Table 3. Although the computational efficiency of these models is relatively high, the mathematical expressions are too simple. They can only indirectly consider the effects of physical properties and stress states of the soil on permanent deformation through regression coefficients, making it difficult to fit the actual situation perfectly.

The empirical regression model was developed to compare against the artificial-neural-network-based prediction model. The empirical regression model adopted in this study is the University of Illinois at Urbana-Champaign (UIUC) rutting model, which is reportedly applicable to coarse-grained granular soils [6,51,52], as shown in Equation (7). The main advantage of this model is that it can adequately consider the impact of the stress level applied to the specimen on permanent deformation. In addition, the model directly considers the shear strength of the test material by introducing the $\frac{\tau_{f}}{\tau_{\max}}$ term.

(7) $$\varepsilon_{p}=AN^{B}\sigma_{d}{}^{c}{\left(\frac{\tau_{f}}{\tau_{\max}}\right)}^{D}=AN^{B}\sigma_{d}{}^{c}{\mathrm{SSR}}^{D}$$

where $\varepsilon_{p}$ is the cumulative plastic strain; N denotes the number of loading cycles;$\;\sigma_{d}$ is the applied deviatoric stress; $\tau_{f}$ is the shear stress on the failure surface; $\tau_{\max}$ is the shear strength; SSR is the shear stress ratio; and *A*, *B*, *C* and *D* are the fitting coefficients.

Table 4 shows the fitted model coefficients for the 60 different sets of test conditions. The majority of the determination coefficients R^2^ are greater than 0.9, indicating that the UIUC model’s fitting results are satisfactory. It should be noted that the model does not account for the impact of moisture content, particle shape and stress history on the accumulation of permanent deformation.

### 4.2. Development of Neural-Network-Based Prediction Model

Machine learning has been gaining popularity in the field of geotechnical engineering as a result of its capability to mine the underlying relationship between multi-source data without any prior assumptions as well as the obvious complexity and uncertainty of the problems in this field. The artificial neural network, a popular machine-learning tool, has been successfully used to predict the mechanical properties of heterogeneous and anisotropic materials, such as soils and aggregates subjected to loading [53]. Compared with empirical models, which may deviate from the actual situation, a more accurate model for predicting the permanent deformation of unbound aggregate base material was constructed based on the back-propagation neural network, which can account for the effects of various factors.

**Figure 11:**
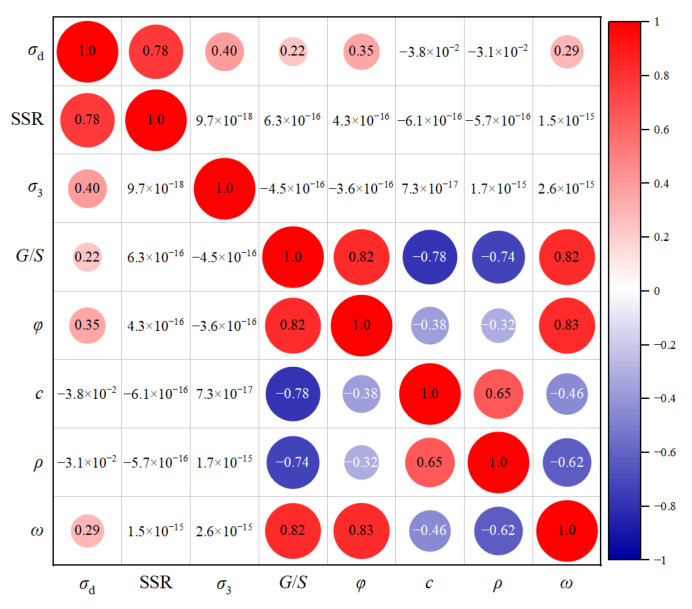
The correlation plot for different input variables.

Among the influencing factors of permanent deformation accumulation studied in the laboratory repeated load triaxial tests, the number of load applications (*N*), shear stress ratio (SSR), confining pressure ($\sigma_{3}$, kPa) and the gradation parameter (*G*/*S*) were selected for use as the input variables of the ANN model, whereas the accumulated plastic axial strain (PD, %) was the only output variable. The statistical parameters of the input and output variables are listed in Table 5. Figure 11 shows the matrix plot of intercorrelations among the different input variables, where the size and color of the circles represent the magnitude of the calculated Pearson correlation coefficient. It should be noted that the influences of both applied stress level and shear strength properties on permanent deformation accumulation were considered in the regression model developed in Section 4.1 through the variable SSR (which is defined as the ratio of applied shear stress to shear strength under the same confining pressure). To improve the predictive performance of the ANN-based model and to avoid overfitting, the confining pressure and gradation parameters were also included as input variables, while the deviatoric stress (which can be back-calculated from the SSR and confining pressure) was not included as an input variable. In addition, the dry density and moisture content were not selected as input variables because all the specimens had the same degree of compaction and were tested at their respective optimal moisture content in this study, which was highly correlated with the *G*/*S* values.

It is necessary to consider the problem of overfitting to develop an artificial neural network model. When a model is overfitted, it performs well on the training data but deteriorates when new data are introduced into the established model, leading to high error values [54,55]. To reduce the complexity of the model and avoid overfitting, the network size should be carefully selected in addition to collecting more training data, as well as using appropriate input variables [56,57]. In this study, certain criteria proposed in the literature were adopted to prevent the network from being overfitted, such as dividing the entire dataset into training and testing sets according to the pre-determined proportions of 80% and 20% [54,58,59,60], respectively. In addition, the ratio of the number of training samples to the number of free parameters should be at least greater than 30 [61]. The training set was used to develop the ANN model, while the testing set was used to assess and verify the generalization ability of the developed model, i.e., how well the model performed on unseen (or new) data. In order to reduce the training time, the training dataset was created by using only the accumulated plastic axial strain data recorded upon a specific number of load applications *N* = 10*x* (where *x* = 1, 2, 3, …, 5000) for each combination of laboratory repeated load triaxial test conditions. As such, the number of training samples was 240,000, which is much more than 30 times the number of weight and bias values.

The performance of ANN is also greatly influenced by the number of hidden layers, the number of hidden neurons and the kind of activation function. Hornik et al. [27] proved that a standard back-propagation network with a sufficient number of hidden neurons can approximate any measurable function to any desired accuracy with a single hidden layer. The logsig function or tansig function is usually selected as the activation function between the input layer and the hidden layer, while a linear function, such as purelin, is mostly used between the hidden layer and the output layer [58]. As an optimization algorithm with good convergence speed and stability, the Levenberg–Marquardt algorithm is usually used to solve nonlinear minimization problems and is suitable for training medium-sized artificial neural networks [57]. Therefore, an artificial neural network with one hidden layer was developed using MATLAB R2021b and the training function trainlm and the learning function learngdm were selected to train the network. Finally, the mean square error (MSE), root mean square error (RMSE), mean absolute error (MAE), as well as coefficient of determination (R^2^) were utilized to evaluate the prediction accuracy of the developed ANN, as shown in Equations (8)–(11). In order to obtain a satisfactory ANN-based model and to improve the prediction accuracy of the model as much as possible, the effects of the aforementioned two activation functions, whether the input and output data are normalized and the number of hidden neurons on the prediction results were discussed in detail, respectively.

(8) $$\mathrm{MSE}=\frac{1}{n}\sum_{i=1}^{n}{\left({\mathrm{PD}}_{\mathrm{meas}}-{\mathrm{PD}}_{\mathrm{pred}}\right)}^{2}$$

(9) $$\mathrm{RMSE}=\sqrt{\frac{\sum_{i=1}^{n}{\left({\mathrm{PD}}_{\mathrm{meas}}-{\mathrm{PD}}_{\mathrm{pred}}\right)}^{2}}{n}}$$

(10) $$\mathrm{MAE}=\frac{1}{n}\overset{n}{\underset{i=1}{\sum }}\left|{\mathrm{PD}}_{\mathrm{meas}}-{\mathrm{PD}}_{\mathrm{pred}}\right|$$

(11) $$R^{2}=1-\frac{\sum_{i=1}^{n}{\left({\mathrm{PD}}_{\mathrm{meas}}-{\mathrm{PD}}_{\mathrm{pred}}\right)}^{2}}{\sum_{i=1}^{n}{\left({\mathrm{PD}}_{\mathrm{meas}}-\bar{{\mathrm{PD}}_{\mathrm{meas}}}\right)}^{2}}$$

where ${\mathrm{PD}}_{\mathrm{meas}}$ is the laboratory-measured values of permanent deformation; ${\mathrm{PD}}_{\mathrm{pred}}$ is the predicted values of permanent deformation obtained from the ANN-based model; $\bar{{\mathrm{PD}}_{\mathrm{meas}}}$ is the mean value of ${\mathrm{PD}}_{\mathrm{meas}}$; n is the number of predicted values.

### 4.3. Optimization of Artificial Neural Network Structure

**Figure 12:**
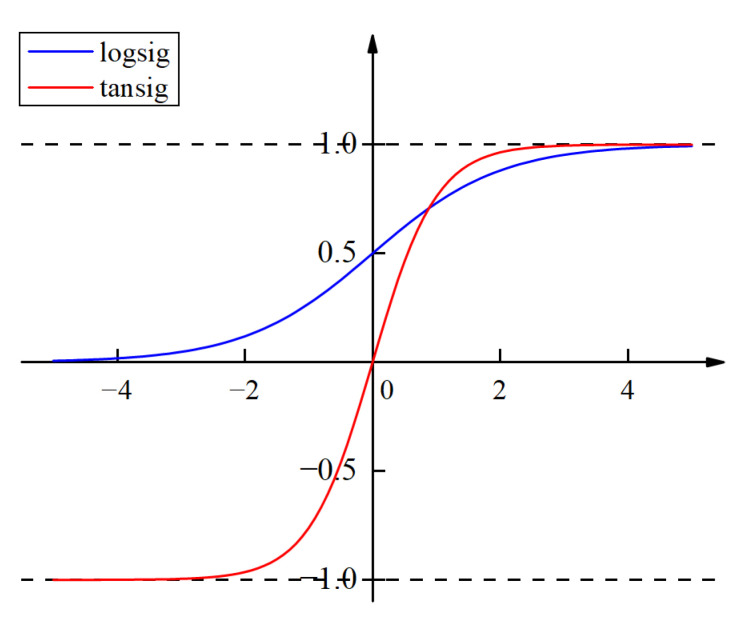
Nonlinear activation function.

The distribution of most data is nonlinear, while the calculation in general neural networks is linear. The purpose of introducing an activation function is to introduce nonlinearity into the neural network to strengthen the learning ability of the network. Figure 12 shows the graphs of logistic function logsig and hyperbolic tangent function tansig. It can be seen that the value of the logsig function ranges from 0 to 1, while the value of the tansig function ranges from −1 to 1. In addition, the tansig function is zero-centered and has a larger gradient compared to the logsig function.

Table 6 compares the prediction accuracy of the ANN-based models using logsig and tansig as the activation function between the input layer and the hidden layer, respectively. It can be seen that the ANN model with the tansig type activation function exhibits higher prediction accuracy.

Data normalization (also called dimensionless normalization) usually maps the data values into intervals [0,1] or [−1,1] to make each input variable have the same influence weight on the prediction results, which is conducive to improving the convergence speed and computational accuracy of the iteration. Before training the network, the built-in function mapminmax of MATLAB was used to normalize the values of the input and output to the interval [0,1], as shown in Equation (12).

(12) $$y=\left(y_{\max}-y_{\min}\right)\times \frac{x-x_{\min}}{x_{\max}-x_{\min}}+y_{\min}$$

where y is the normalized value; x is the original value before normalization; $y_{\min}$ is the minimum value of the normalized interval and is set to 0; $y_{\max}$ is the maximum value of the normalized interval and is set to 1; $x_{\min}$ is the minimum value before normalization; $x_{\max}$ is the maximum value before normalization.

Table 7 compares the effect of data normalization on the predictive performance of the ANN-based model. It is clear that data normalization improves the prediction accuracy of the ANN-based model. Although the Levenberg–Marquart algorithm (trainlm in MATLAB) is less dependent on normalization than the gradient descent method (trainlm in MATLAB), normalization can initialize each neuron into an effective state, that is, the neurons can effectively utilize the nonlinear part of the activation function to prevent the output saturation and ensure that the input variables with small values are not ignored.

**Figure 13:**
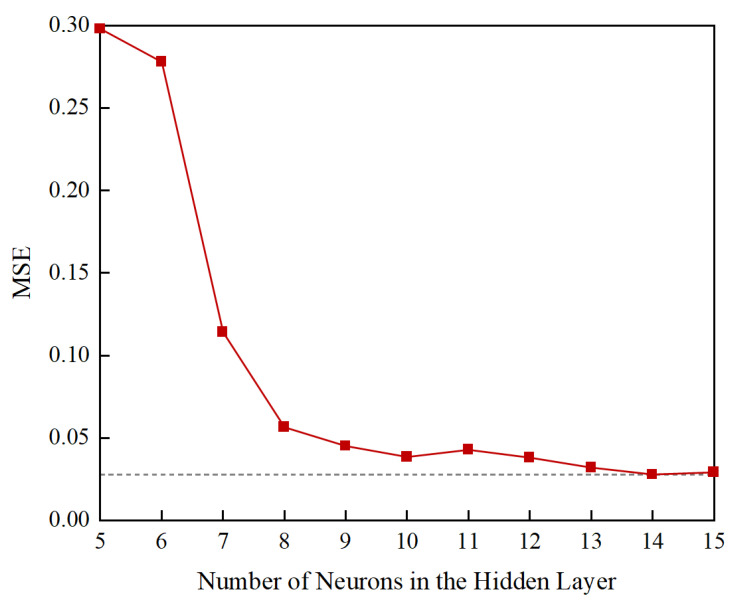
The effect of the number of hidden neurons on the prediction accuracy of the ANN-based model.

**Figure 14:**
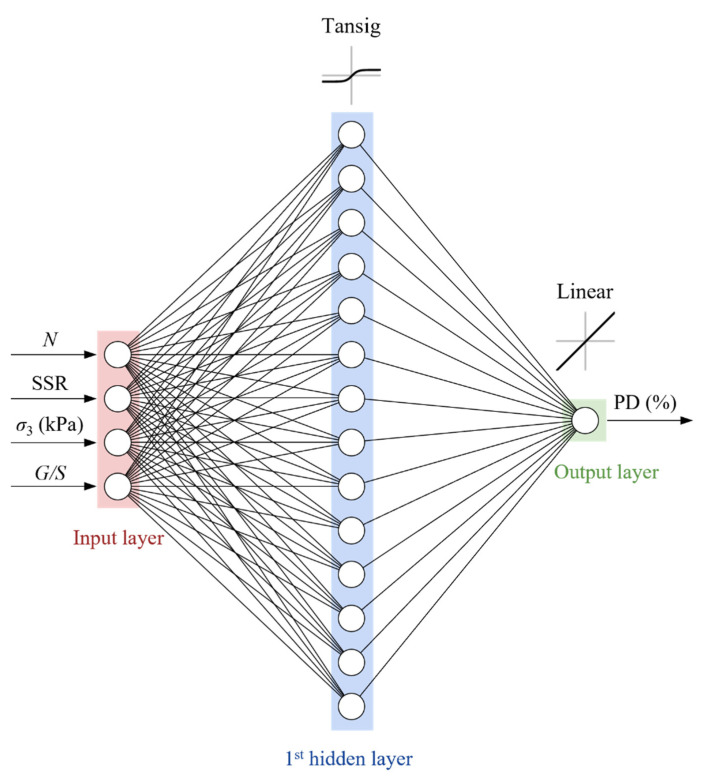
The ANN-based prediction model with the optimal network topology.

In addition to the activation function and the characteristics of the data, the number of hidden neurons also affects the performance of the neural network, which needs to be carefully selected depending on the complexity of the problem and the size of the dataset. Several ANN with different numbers of hidden neurons were tested to develop an ANN-based model with the optimal network topology for predicting the permanent deformation of UGMs. The mean square error was used to evaluate the prediction accuracy of the developed models. The results of the parameterization study are shown in Figure 13. It can be seen that the ANN model with 14 hidden neurons demonstrates the best predictive performance. Therefore, this model was selected as the optimal prediction model with the network structure shown in Figure 14.

The prediction accuracy of the optimal ANN-based model on the training set, testing set and all datasets is summarized in Table 8. The coefficient of determination R^2^ is equal to 0.999 for both training and testing sets, which indicates that the established ANN-based model has a strong ability to predict permanent deformation. The mean absolute error value MAE for the testing set is 0.0406, indicating that the model is well trained and has a satisfactory generalization ability. In addition, the performance parameters of the model on the training and testing sets are very close, and this indicates that the model does not suffer from overfitting.

**Figure 15:**
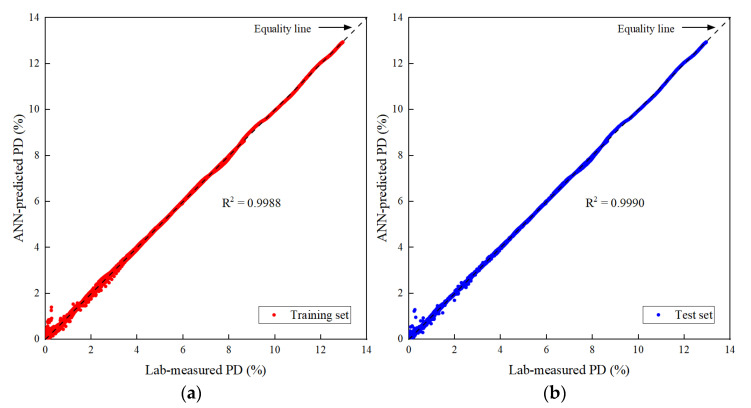
The goodness-of-fit results: (**a**) Training set; and (**b**) Testing set.

The ANN-predicted ${\mathrm{PD}}_{\mathrm{pred}}$ were plotted against the laboratory-measured ${\mathrm{PD}}_{\mathrm{meas}}$ for both training and testing sets. As shown in Figure 15, there is a fairly good alignment between the ANN-estimated ${\mathrm{PD}}_{\mathrm{pred}}\;$and the laboratory-measured ${\mathrm{PD}}_{\mathrm{meas}}$. Both the training and testing results indicate a fairly high accuracy of the ANN model in estimating PD based on the physical properties and the applied stress level.

## 5. Discussion

### 5.1. Comparison of the Predicted and Actual Values of Permanent Deformation

**Figure 16:**
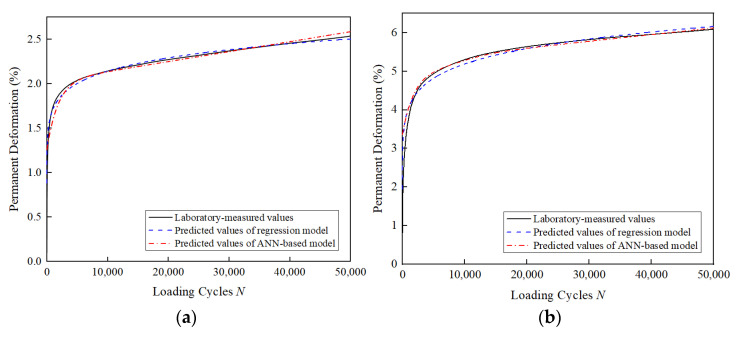

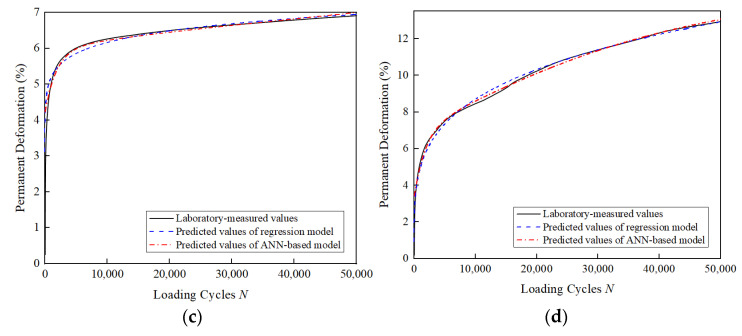
Comparison between the predicted and measured permanent deformation: (**a**) *G*/*S* = 2.5, $\sigma_{3}$ = 50 kPa, SSR = 0.7; (**b**) *G*/*S* = 2.5, $\sigma_{3}$ = 50 kPa, SSR = 0.9; (**c**) *G*/*S* = 2.5, $\sigma_{3}$ = 150 kPa, SSR = 0.7; (**d**) *G*/*S* = 2.5, $\sigma_{3}$ = 150 kPa, SSR = 0.9.

Figure 16 compares the predicted values of permanent deformation obtained from the developed ANN-based model and the regression model with the laboratory-measured values for some of the test conditions. It can be observed that the ANN-based model can successfully reproduce the cumulative plastic deformation behavior of the unbound aggregate base materials during the test, and the predicted values of the model are very close to the laboratory-measured values.

The above comparisons show that the ANN-based model outperforms the empirical model in terms of predictive performance. The findings also agree well with previous studies demonstrating that artificial neural networks have better predictive performance than empirical models. One possible explanation for this phenomenon is that artificial neural networks are able to model the complex nonlinear relationships between inputs and outputs with the help of the neurons in the hidden layer. In contrast, empirical models can only make predictions based on pre-specified, limited statistical relationships.

### 5.2. Sensitivity Analysis

Neural networks are less interpretable than empirical models with explicit expressions and are often described as “black box” models. For ANNs, it is not easy to show an explicit relationship between the inputs and outputs. In general, the more parameters the input has, the better the model prediction results will be. However, when the inputs have redundant or irrelevant features, the prediction accuracy of the network is likely to decrease [62]. The Garson algorithm [63] was used to evaluate the degree of influence of the number of loading cycles *N*, shear stress ratio SSR, confining pressure $\sigma_{3}$ and gradation parameter *G*/*S* on permanent deformation, as shown in Equation (13).

(13) $$\mathrm{IC}=\frac{\sum_{j=1}^{n\;\mathrm{hidden}}\frac{\left|w_{ji}\times w_{oj}\right|}{\sum_{I=1}^{n\;\mathrm{inputs}}\left|w_{jI}\right|}}{\sum_{k=1}^{n\;\mathrm{inputs}}\left(\sum_{j=1}^{n\;\mathrm{hidden}}\frac{\left|w_{jk}\times w_{oj}\right|}{\sum_{I=1}^{n\;\mathrm{inputs}}\left|w_{jI}\right|}\right)}$$

where IC is the influence coefficient of the input variable to the output value; $w_{ji}$ is the weight between the *j*th hidden neuron and the ith input neuron; $w_{oj}$ is the weight between the output neuron and the *j*th hidden neuron; i, k, I represent the input neuron; j and o represent the hidden neuron and the output neuron, respectively.

**Figure 17:**
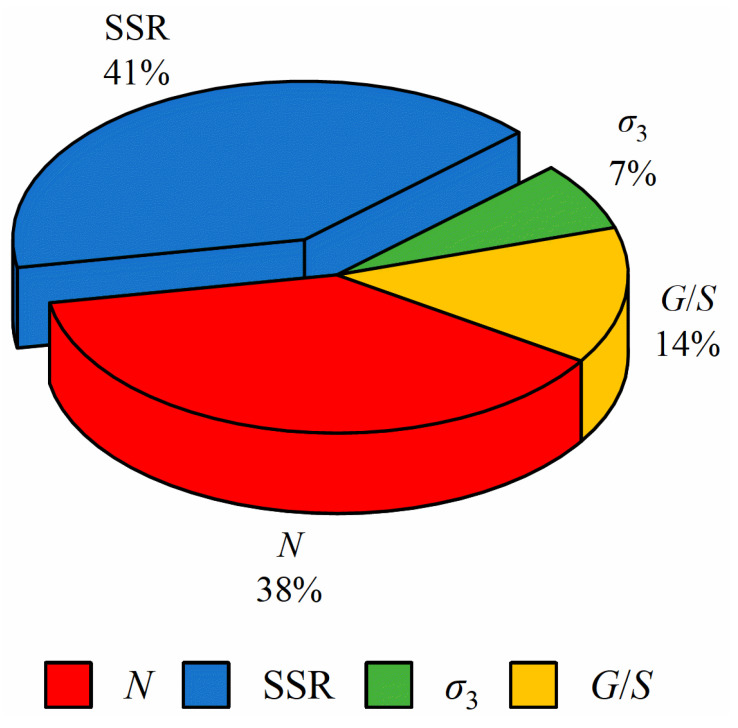
Influence coefficients of the selected input variables of the predicted permanent deformation.

Table 9 lists the weight and bias values between the neurons in the neighboring layer in the optimal ANN. The influence coefficients of the input variables to the predicted permanent deformation are calculated according to Equation (13) and shown in Figure 17. It can be seen that all the selected input variables are related to the output variable of accumulated plastic axial strain. Among those input variables, the shear stress ratio (SSR) has the greatest influence on the output variable, followed by the number of load applications (*N*). The influence of confining pressure on the accumulated plastic strain is less than that of gradation, i.e., the influence coefficient of the former is only half that of the latter. These findings are consistent with the experimental results described in Section 3. Therefore, during the process of road construction and maintenance, not only the aggregates with high shear strength and good gradation should be selected as base materials, but the preventive maintenance of the pavement should also be strengthened, and the overloaded vehicles should be strictly controlled.

## 6. Summary and Conclusions

Research efforts were made to develop an artificial-neural-network (ANN)-based model to predict the permanent deformation accumulation of unbound granular materials (UGMs). The ANN model was first trained and further validated by using the accumulated plastic axial strain data recorded from laboratory repeated load triaxial tests. The main conclusions drawn from this study were summarized as follows:

- The consideration of both the shear stress ratio and deviatoric stress (or equivalently, confining pressure) is regarded indispensable for investigating the permanent deformation accumulation characteristics of UGMs. The applied stress level affects not only the magnitude but also the accumulation pattern of the permanent deformation induced. The effects of confining pressure and gradation on the accumulated plastic strain are more significant at a high shear stress ratio (e.g., SSR ≥ 0.7) than those at a relatively low shear stress ratio (e.g., SSR ≤ 0.5).
- Gradation has a remarkable effect on the long-term plastic strain accumulation of UGMs under repeated loading due to different inherent packing structures. The accumulated plastic axial strain was clearly the smallest for UGMs with gradation parameter *G*/*S* = 1.6 under all stress states of repeated loading, followed by UGMs with *G*/*S* = 1.0 and *G/S* = 2.5.
- The artificial-neural-network (ANN)-based model was proposed for predicting the permanent deformation accumulation of UGMs. It was found that the ANN-based model has better prediction accuracy and generalization ability than the classic empirical models.
- The ANN model with an input layer containing 4 neurons, a hidden layer containing 14 neurons and accumulated permanent deformation as the single output was constructed. The coefficients of determination for both the training and testing datasets were greater than 0.99, and the minimum mean square error value of the network was 0.0042. The sensitivity analysis results reveal that the number of load applications (*N*) and shear stress ratio (SSR) are the most critical factors influencing the permanent deformation accumulation of UGMs, while the gradation and confining pressure also play important roles.
- The ANN-based model developed in this study can be used to predict the accumulated plastic deformation of unbound aggregate base courses under long-term applications of repeated traffic loading, thus providing the theoretical basis for improving their structural design and deformation performance assessment.

It is worth noting that the empirical models usually need to be calibrated for different testing conditions, while developing ANN models requires a careful selection of optimization functions and training parameters. The major advantage of ANN-based models over empirical models is that the former can predict the accumulated plastic deformation of unbound aggregate base materials with various combinations of gradation and stress states (e.g., quantified by confining pressure and shear stress ratio) at any number of load applications by using only one single set of parameters. However, the empirical models rely on pre-specified equations incorporating these independent variables to make predictions, and the development of such equations with an acceptable prediction accuracy is troublesome and tedious. Therefore, the ANN-based model would be a better alternative.

## Figures and Tables

**Table 1:** The designed gradation and related parameters.

*G*/*S*	*D*_max_ (mm)	*n*	*C*_u_	*C*_c_
1.0	26.5	0.32	270.22	3.55
1.6	26.5	0.51	29.09	1.97
1.8	26.5	0.56	23.64	2.08
2.0	26.5	0.61	18.09	1.90
2.5	26.5	0.71	11.63	1.63

Notes: *C*_u_ is the coefficient of uniformity, and *C*_c_ is the coefficient of curvature.

**Table 2:** The testing matrix of laboratory repeated load triaxial tests.

Specimen No.	*G*/*S*	$\sigma_{3}\;(\mathrm{kPa})$	$\sigma_{d}\;(\mathrm{kPa})$	φ (°)	c (kPa)	$\rho_{\max}\;(g/{\mathrm{cm}}^{3})$	$\omega_{\mathrm{opt}}\;(\%)$
A050	1.0	50	71/128/195/273	29.2	65.5	2.341	4.311
A100		100	93/166/253/355				
A150		150	114/205/311/437				
B050	1.6	50	97/178/277/400	40.9	64.8	2.344	4.329
B100		100	136/250/288/560				
B150		150	175/321/498/720				
C050	1.8	50	128/236/370/542	47.9	64.3	2.351	4.470
C100		100	184/341/535/783				
C150		150	241/446/699/1024				
D050	2.0	50	106/195/304/441	43.6	64.0	2.271	4.481
D100		100	152/277/432/628				
D150		150	196/360/561/815				
E050	2.5	50	94/173/271/394	45.5	48.7	2.270	4.472
E100		100	142/261/407/592				
E150		150	189/348/543/790				

Notes: φ is the internal friction angle; c is the apparent cohesion; $\rho_{\max}$ is the maximum dry density; and $\omega_{\mathrm{opt}}$ is the optimum moisture content.

**Table 3:** Commonly used regression models for permanent deformation prediction.

Model No.	Model Equations	Authors
1	$\varepsilon_{p}=a\left(1+b\log N\right)$	Barksdale [44]
2	$\varepsilon_{p}=aN^{b}$	Monismith et al. [16]
3	$\varepsilon_{p}=a\left[1-{\left(\frac{N}{100}\right)}^{-b}\right]$	Hornych et al. [45]
4	$\varepsilon_{p}=\left(cN+a\right)\left(1-e^{-bN}\right)$	Wolff and Visser [46]
5	$\varepsilon_{p}=aN^{b}$+$c\left(e^{-dN}-1\right)$	Huurman [47]
6	$\varepsilon_{p}=CN^{b}\frac{R}{A-R}$	Korkiala [48]
7	$\varepsilon_{p}=a\left(1-e^{-bN}\right)$	Li et al. [49]
8	$\varepsilon_{p}=\frac{N^{b}}{a+cN^{b}}$	Ren et al. [50]

Notes: $\varepsilon_{p}$ is the cumulative plastic strain, *N* is the number of loading cycles, and the remaining symbols in the table are the model coefficients to be fitted.

**Table 4:** Fitted model coefficients of the UIUC rutting model.

*G*/*S*	$\sigma_{d}$	SSR	A	B	C	D	RMSE	R^2^
1.0	50	0.3	0.4832	0.0684	0.9220	4.1788	0.0051	0.9411
		0.5	0.2505	0.0849	0.8005	5.0295	0.0133	0.9635
		0.7	0.1888	0.0534	0.7651	6.4989	0.0342	0.8785
		0.9	0.0982	0.1169	0.6790	17.1183	0.0305	0.9849
	100	0.3	0.4649	0.0819	0.9136	4.2107	0.0072	0.9515
		0.5	0.2729	0.0653	0.8210	4.9240	0.0207	0.9218
		0.7	0.1764	0.0757	0.7623	6.6560	0.0483	0.9247
		0.9	0.1265	0.0832	0.7246	15.1815	0.0459	0.9795
	150	0.3	0.5285	0.0751	0.9420	4.1003	0.0126	0.9382
		0.5	0.2882	0.0587	0.8338	4.8561	0.0293	0.8934
		0.7	0.1731	0.0698	0.7653	6.7009	0.0522	0.9141
		0.9	0.1119	0.1449	0.7162	16.0544	0.0821	0.9908
1.6	50	0.3	0.3816	0.0839	0.8731	4.3648	0.0041	0.9585
		0.5	0.2456	0.0601	0.8050	5.0533	0.0186	0.8794
		0.7	0.1248	0.0956	0.7142	7.4552	0.0179	0.9745
		0.9	0.0784	0.1024	0.6632	18.8969	0.0167	0.9873
	100	0.3	0.4277	0.0719	0.8980	4.2769	0.0078	0.9299
		0.5	0.2117	0.0807	0.7896	5.2340	0.0171	0.9494
		0.7	0.1301	0.0617	0.7313	7.3654	0.0264	0.9022
		0.9	0.0922	0.1121	0.6983	17.6156	0.0418	0.9874
	150	0.3	0.4356	0.0644	0.9026	4.2622	0.0151	0.8175
		0.5	0.2020	0.0942	0.7886	5.2898	0.0179	0.9734
		0.7	0.1169	0.0931	0.7251	7.6077	0.0263	0.9709
		0.9	0.0944	0.1091	0.7096	17.4442	0.0341	0.9947
1.8	50	0.3	0.3911	0.0718	0.8806	4.3455	0.0040	0.9634
		0.5	0.1988	0.0902	0.7787	5.3089	0.0125	0.9709
		0.7	0.1376	0.0689	0.7377	7.2348	0.0229	0.9549
		0.9	0.0810	0.0893	0.6797	18.6554	0.0258	0.9760
	100	0.3	0.3638	0.0857	0.8709	4.4003	0.0068	0.9564
		0.5	0.2009	0.0752	0.7891	5.2971	0.0222	0.9225
		0.7	0.1123	0.1107	0.7224	7.6945	0.0216	0.9888
		0.9	0.0808	0.1225	0.6934	18.6340	0.0526	0.9849
	150	0.3	0.3954	0.0778	0.8872	4.3376	0.0121	0.9298
		0.5	0.1982	0.0823	0.7931	5.3137	0.0178	0.9740
		0.7	0.1038	0.1158	0.7208	7.8728	0.0417	0.9692
		0.9	0.3426	0.0829	0.8214	7.2148	0.0627	0.9738
2.0	50	0.3	0.4141	0.0869	0.8901	4.3025	0.0060	0.9572
		0.5	0.2259	0.0777	0.7936	5.1556	0.0164	0.9412
		0.7	0.1528	0.0813	0.7466	6.9900	0.0297	0.9639
		0.9	0.0989	0.0805	0.6983	17.1070	0.0442	0.9498
	100	0.3	0.4260	0.0753	0.8977	4.2802	0.0088	0.9358
		0.5	0.4697	0.0817	0.9135	4.2193	0.0186	0.9573
		0.7	0.1592	0.0799	0.7624	6.8952	0.0605	0.9357
		0.9	0.1102	0.0722	0.7243	16.2673	0.0797	0.9321
	150	0.3	0.4271	0.0938	0.8992	4.2787	0.0146	0.9487
		0.5	0.1568	0.1550	0.7568	5.5818	0.0298	0.9716
		0.7	0.1496	0.0851	0.7608	7.0390	0.0607	0.9543
		0.9	0.0823	0.1744	0.6990	18.3691	0.1142	0.9889
2.5	50	0.3	0.4597	0.0782	0.9112	4.2198	0.0059	0.9593
		0.5	0.2305	0.0865	0.7940	5.1305	0.0180	0.9457
		0.7	0.1574	0.0967	0.7476	6.9182	0.0285	0.9807
		0.9	0.1252	0.1066	0.7272	15.2272	0.1315	0.9460
	100	0.3	0.4363	0.0747	0.9019	4.2615	0.0077	0.9489
		0.5	0.1993	0.1086	0.7819	5.3036	0.0226	0.9627
		0.7	0.1920	0.0773	0.7867	6.4585	0.0599	0.9694
		0.9	0.1171	0.0859	0.7305	15.7838	0.0591	0.9822
	150	0.3	0.4994	0.0669	0.9284	4.1512	0.0203	0.8775
		0.5	0.2303	0.0737	0.8092	5.1317	0.0441	0.8601
		0.7	0.1991	0.0740	0.7977	6.3732	0.1266	0.9279
		0.9	0.0709	0.2455	0.6851	19.1834	0.1593	0.9941

Note: R^2^ is the coefficient of determination, and RMSE is the root mean square error.

**Table 5:** Statistical parameters of the input and output variables used in this study.

Statistical Parameters	Input Variables				Output Variable
	*N*	SSR	$\sigma_{3}$	*G*/*S*	PD
Maximum	50,000	0.9	150	2.5	12.9555
Minimum	10	0.3	50	1.0	0.0060
Median	25,005	0.6	100	1.8	1.4535
Mean	25,005	0.6	100	1.7800	2.2008
Standard deviation	14,435.2	0.2582	50	0.5495	2.0921
Coefficient of variance	1.7322	2.3238	2	3.2390	1.0520

**Table 6:** The effect of two different activation functions on the predictive performance of the ANN-based model.

Performance Parameters	Activation Function	
	logsig	tansig
MSE (mm^2^)	0.0051	0.0043
RMSE (mm)	0.0717	0.0657
MAE (mm)	0.0448	0.0427
R^2^	0.9988	0.9990

**Table 7:** The effect of data normalization on the predictive performance of the ANN-based model.

Performance Parameters	Data Normalization	
	Without	With
MSE (mm^2^)	0.0072	0.0043
RMSE (mm)	0.0846	0.0657
MAE (mm)	0.0490	0.0427
R^2^	0.9984	0.9990

**Table 8:** The results of prediction accuracy indices of the optimal ANN-based model.

Performance Parameters	Dataset		
	Training Set	Testing Set	Overall
MSE (mm^2^)	0.0053	0.0049	0.0052
RMSE (mm)	0.0728	0.0703	0.0723
MAE (mm)	0.0406	0.0406	0.0406
R^2^	0.9988	0.9989	0.9988

**Table 9:** The weight and bias values of the developed ANN-based model.

Number of Hidden Neurons (*j*)	$w_{j1}$	$w_{j2}$	$w_{j3}$	$w_{j4}$	$w_{oj}$	$\theta_{ji}\;$
1	−0.113	−4.191	−0.399	0.784	−58.986	7.901
2	−2.597	87.644	−8.837	−28.461	−0.110	−70.350
3	0.000	0.658	0.908	0.469	0.239	−0.183
4	0.190	−7.038	−22.867	−9.040	0.074	6.952
5	−0.023	0.572	0.075	1.297	−4.016	−2.775
6	−0.962	−12.145	−3.371	−4.020	−0.625	18.961
7	−0.038	−1.147	1.285	−1.047	−0.108	0.922
8	−0.177	3.441	0.648	−5.559	−0.070	−0.770
9	0.064	2.339	0.537	3.224	−5.314	−3.012
10	−0.516	5.357	6.681	−3.762	3.493	−4.368
11	7.763	−0.486	−0.217	−0.153	56.515	11.303
12	−0.032	−0.660	−0.033	1.477	−0.205	0.948
13	0.519	−5.267	−6.590	3.717	3.482	4.310
14	−0.057	−2.180	−0.505	−2.951	−6.094	2.835

Notes: $\theta_{ji}$ are the bias values between the input layer and the hidden layer, $\theta_{oj}$ is the bias value between the hidden layer and the output layer, and $\theta_{oj}=-0.8491$.

## Data Availability

Not applicable.
